# Contact osteogenesis by biodegradable 3D-printed poly(lactide-co-trimethylene carbonate)

**DOI:** 10.1186/s40824-022-00299-x

**Published:** 2022-10-10

**Authors:** Mohamad Nageeb Hassan, Mohammed Ahmed Yassin, Ahmed Maher Eltawila, Ahmed Emad Aladawi, Samih Mohamed-Ahmed, Salwa Suliman, Sherif Kandil, Kamal Mustafa

**Affiliations:** 1 Department of Clinical Dentistry, Faculty of Medicine, Centre for Translational Oral Research (TOR), University of Bergen, Årstadveien 19, 5009 Bergen, Norway; 2 Department of Materials Science, Institute of Graduate Studies and Research (IGSR), Alexandria University, El-Shatby, Alexandria, 21526 Egypt; 3 Department of Dental Biomaterials, Faculty of Oral and Dental Medicine, Delta University for Science and Technology, Coastal International Road, Gamasa, 11152 Egypt; 4 Department of Dental Biomaterials, Faculty of Dentistry, Alexandria University, El-Azarita, Alexandria, 21526 Egypt

**Keywords:** 3D-printing, Poly(lactide-co-trimethylene carbonate), Polycaprolactone, Printability, Degradation, ALP activity, Osteoconduction

## Abstract

**Background:**

To support bone regeneration, 3D-printed templates function as temporary guides. The preferred materials are synthetic polymers, due to their ease of processing and biological inertness. Poly(lactide-co-trimethylene carbonate) (PLATMC) has good biological compatibility and currently used in soft tissue regeneration. The aim of this study was to evaluate the osteoconductivity of 3D-printed PLATMC templates for bone tissue engineering, in comparison with the widely used 3D-printed polycaprolactone (PCL) templates.

**Methods:**

The printability and physical properties of 3D-printed templates were assessed, including wettability, tensile properties and the degradation profile. Human bone marrow-derived mesenchymal stem cells (hBMSCs) were used to evaluate osteoconductivity and extracellular matrix secretion in vitro. In addition, 3D-printed templates were implanted in subcutaneous and calvarial bone defect models in rabbits.

**Results:**

Compared to PCL, PLATMC exhibited greater wettability, strength, degradation, and promoted osteogenic differentiation of hBMSCs, with superior osteoconductivity. However, the higher ALP activity disclosed by PCL group at 7 and 21 days did not dictate better osteoconductivity. This was confirmed in vivo in the calvarial defect model, where PCL disclosed distant osteogenesis, while PLATMC disclosed greater areas of new bone and obvious contact osteogenesis on surface.

**Conclusions:**

This study shows for the first time the contact osteogenesis formed on a degradable synthetic co-polymer. 3D-printed PLATMC templates disclosed unique contact osteogenesis and significant higher amount of new bone regeneration, thus could be used to advantage in bone tissue engineering.

**Supplementary Information:**

The online version contains supplementary material available at 10.1186/s40824-022-00299-x.

## Introduction

Extensive work has been introduced through bone tissue engineering (BTE) to replace the current treatment options for augmentation/replacement of lost bone tissues, circumventing the limitations associated with autogenous, allogenic, or xenogeneic grafts [1]. In addition to the classical requirements of biocompatibility, tailored biodegradation rate, adequate mechanical properties, porosity, sterilizability and off-the-shelf availability, the ideal template for BTE should offer adequate osteoconductivity [2].

Osteoconduction is defined as the ability to support recruitment and migration of differentiating osteogenic cells to the implanted surface. The implanted surface should promote osteogenic cell activation and extracellular matrix (ECM) deposition to allow for the next healing phase known as new (de novo) bone formation directly on its surface [3]. The combination of these two healing phases results in contact osteogenesis, at the light microscopic level, this appears as intimate bone contact to the implanted surface, commonly known as osseointegration [4].

At the ultrastructural level of contact osteogenesis, the collagen compartment of the bone is separated from the implanted surface by a continuous submicron-thick layer involving individual fused globules known as globular accretions, forming the cement line matrix [5, 6]. Approximately 1 µm diameter, these globular accretions were first described by the group of John Davies, in the early 90’s [7], as the primary event in mineralized ECM secretion by active (secretory) osteoblasts on implanted materials, before the deposition of overlying mineralizing collagen matrix [6]. In contrast, bone could be formed in relation to implanted materials through distance osteogenesis, similar to physiologic appositional bone growth, that encroaches on the implant surface. Hence, the bioinert (non-osteoconductive) implant becomes surrounded by bone through distance osteogenesis, but always partially obscured by general fibrous connective tissue ECM [5].

The biologically-derived natural polymers are considered biologically active, with osteoconductive properties. However, they are characterized by suboptimal mechanical properties and questions have been raised about their tissue reactivity and purification complexity [8, 9]. In contrast, biodegradable synthetic polymers used in BTE tend to be bioinert and incapable of performing specific biological functions [10]. They offer the advantage of mechanical strength, resilience, and ease of processing. However to date, there are no reports of synthetic polymers exhibiting inherent osteoconductivity which activates contact osteogenesis on the surface [11]. Thus, many attempts were further applied to boost their physical properties and osteoconductivity, customized per application, including co-polymerization, blending, making composites and functionalized coatings [12].

Aliphatic polyesters are thermoplastic polymers with hydrolytically degradable aliphatic ester linkages, which have been extensively investigated in BTE applications. Among the most extensively studied are polylactide (PLA), polylactide-co-glycolide (PLGA) and polycaprolactone (PCL).

PCL is a semi-crystalline polymer that is highly processible due to its low melting point (55–60 °C); it usually takes 24 to 36 months before full biodegradation. The first 3D-printed templates introduced for BTE in the calvarial bone defect (CBD) model were fabricated from PCL [13], with following successful clinical trials [14, 15]. Thus, 3D-printed medical-grade PCL templates were approved by FDA for clinical use [12].

In contrast, poly(trimethylene carbonate) (PTMC) are high molecular weight, amorphous polymers (aliphatic polycarbonates which contain a carbonate ester group in their main chain). They exhibit excellent flexibility and surface degradation profile, but poor mechanical strength, and have been investigated as potential implant materials for soft tissue regeneration [16, 17]. Co-polymer networks of PLA with PTMC, known as poly(lactide-co-trimethylene carbonate) (PLATMC), prepared with various PTMC content (mol %), showed higher toughness, flexibility and elongations at break (up to 800%) [18, 19]. In addition, they were found to degrade through bulk hydrolysis autocatalyzed by the generated acidic end groups [20], and have been used to support soft tissue regeneration with excellent biocompatibility [21, 22]. PLATMC was recently used by our group for some BTE applications, and showed positive results within the limitations of the experiments [23, 24].

Promising results have been reported for 3D-printed templates, which are reproducible, highly porous structures with superior interconnectivity [25]. BTE is enhanced through these 3D-printed templates and bone ingrowth was revealed within the strands of the template [26]. The aim of this study was to characterize the osteoconductivity of 3D-printed PLATMC, compared to the widely used PCL, as BTE templates. The degradation of PLATMC has been determined in vitro, by monitoring mass loss and surface erosion according to previously reported protocols [27, 28]. The osteoconductive potential of the printed templates was tested in vitro using human bone marrow-derived mesenchymal stem cells (hBMSC), where cell attachment, proliferation, osteogenic differentiation, and ECM secretion were assessed. This was then evaluated in vivo, where the subcutaneous and CBD models in rabbits were used to evaluate tissue response to the implanted templates and the amount of new bone formation, respectively.

## Methods

### Printing of PCL and PLATMC

**Fig. 1:**
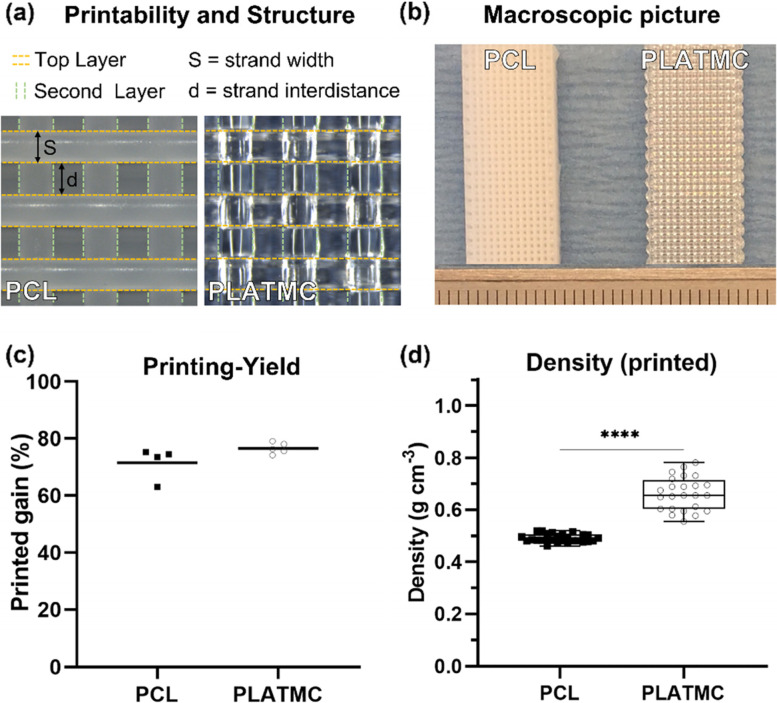
Printability of the 3D-printed PCL and PLATMC, and their calculated printing-yield and density of the printed templates. **a** microscopic pictures to the printed structures, marked with dashed lines to track the strands in the top two layers, on which the strand width (diameter) and strand interdistance were measured to determine the printability of each polymer. **b** macroscopic pictures for the printed structures, scale bar in mm. **c** graph for the mean printing-yield (*n* = 4), and (**d**) box plots for the density of the printed templates (*n* = 25). The statistical significance between the groups is marked with Asterisks (*), **** *p* < 0.0001

Medical grade PCL (RESOMER C 212, Evonik—Germany) and PLATMC (Resomer LT 706 S, Evonik—Germany) were used as received and printed using a pneumatic melting-extrusion printer (3D-Bioplotter, Manufacturer Series, EnvisionTEC, Germany). The printing structure was designed to print strands with 0.4 mm diameter, strand interdistance of 0.35 mm, and 0/90° angle between layers (Fig. 1a).

### Printability and yield calculations

The printability of both polymers was measured through their output shape fidelity. The ratio of the measured printed strand diameter (S) over the measured strand interdistance (d) was calculated and compared to the related ratios in their ideal design (Fig. 1a). In addition, the printing-yield and density of the printed templates were calculated for each polymer, to allow comparison of their processing efficiency. The printing-yield was calculated according to the following equation:

$$\mathrm{Printing}\ \mathrm{Yield}\;\left(\%\right)=\frac{W_{Print}}{{W}_{feed}}\times100$$

where *W*_*print*_ is the total weight of printed templates/each printing-run and *W*_*feed*_ is the gross weight of the feed materials added to the printing cartridge for each specific printing-run. On the other hand, the weight of the printed groups was recorded to calculate their densities (g cm^−3^) as follows: density = *W*_*print*_*/V*_*print*_, where *W*_*print*_ is the weight of printed templates in grams, while *V*_*print*_ is their calculated geometric volume.

### Sterilization of printed templates for biological assessment

All printed templates used for biological characterization (in vitro and in vivo) were prewashed using sterilized 1 × PBS plus sonication (5–10 min, twice) followed by immersion in ethanol (70%, 30 min, twice), then the ethanol was aspirated in a safety cabinet, followed by drying (ethanol full evaporation at room temperature (*RT*)). The templates were then exposed to UV light for 1 h and packed in sterile bags, before removal from the safety cabinet and storage until use.

### Physical and mechanical testing of printed templates

#### Wettability

The water contact angle test was applied (at *RT*) on the prepared blends, either 3D-printed (*n* = 5) or cast into flat sheets (*n* = 3), to determine their hydrophilicity, using (Contact Angle Goniometer Model 90, CA Edition, ramé-hart—USA). Water (3 μL) was dropped onto the surface of each sample and the average contact angle was recorded (for triple measurements) at various positions on the surface.

#### Tensile properties

Dumbbell-shaped samples (shaft dimensions = 17.5 × 4.5 × 1.5 mm) were printed according to ASTM-D638 to test the mechanical properties of each group. The tensile strength, Young's Modulus and elongation at break (*n* = 5) were tested using a universal tensile testing machine (MTS, 858 Mini Bionix II instrument, Eden Prairie, MN, USA), at room temperature, and rate of tensile displacement at 3 mm sec^−1^.

#### Degradation (In vitro)

Printed PCL and PLATMC samples (Ø = 8 mm, *n* = 5) were weighed precisely (*W*_*o*_) then put in PBS (900 µL/sample) in 48 well plates. The wells were coded, to guarantee later matching of their mass change (specific per each sample), sealed, and incubated (37 °C, shaking 100 RPM). The PBS was replaced with a fresh preparation every 5 days, up to 100 days. The mass change was recorded at 15, 30, 60 and 100 days, where the samples were washed (dH_2_O, 3 times) dried at (37 °C, 4 h), frozen (overnight) and freeze-dried (48 h) before being weighed (*W*_*t*_). The Mass loss (%) was calculated according to the following equation:

$$Mass\ loss \left(\mathrm{\%}\right)=\frac{\left(W{\text{o}}-W{\text{t}}\right)}{W{\text{o}}} \times 100$$

where *W*_*o*_ is the original weight of each template before immersion in PBS, and *W*_*t*_ is the dry weight recorded at each time point. In addition, the surface morphology of the tested templates was recorded at three time points, after 1, 60 and 100 days of incubation, using scanning electron microscopy (SEM) (Phenom XL Desktop, Thermo Fisher). The templates were dried and then sputter coated with gold‑platinum (around 50 Ångstrom thickness) and scanned by a secondary electron detector.

### *In vitro* osteogenic characterization using hBMSCs

#### Cell seeding and efficiency calculations

After informed parental consent, donated bone marrow aspirates (10 mL) were obtained from the anterior iliac crest of 8–14 years-old patients, undergoing iliac crest surgery for cleft lip and palate repair at the Department of Plastic, Hand and Reconstructive Surgery, National Fire Damage Center, Bergen – Norway. Ethical approval for this study was granted by the Regional Committee for Medical and Health Research Ethics (REK) in Norway (Ref. No. 2013/1248/REK sør-øst C). The hBMSCs were isolated from bone marrow aspirates, characterized according to our protocols [29]. The cells were kept frozen in liquid nitrogen (passage 2), then thawed in α-MEM, expanded, and seeded onto the printed templates. One day after seeding, osteogenic supplements (0.1 mM L-ascorbic acid 2-phosphate, 10 mM β-GP, and 100 nM dexamethasone) were added to the culture medium to provide the essential factors needed for osteogenic differentiation and matrix biomineralization. The culture medium with osteogenic supplements was changed twice weekly.

The seeding efficiency of hBMSCs on printed PCL and PLATMC (2 × 10^5^ cell cm^−2^) was calculated after seeding for 8–12 h, incubated at 37 °C and 5% CO_2_. The seeded templates were then transferred to another plate, and the remaining cells, attached and suspended cells per each well, were collected (1.5 mL Eppendorf safe-lock tubes), centrifuged, resuspended in 100 µL α-MEM, stained (4% trypan blue) and counted. The seeding efficiency was calculated using the following equation:

$$Seeding\;Efficiency\;\left(\%\right)=\frac{\left(\mathrm{Seededcells}-\mathrm{Remainingcells}\right)}{\mathrm{Seededcells}}\times100$$

#### Cytoskeleton immunofluorescence staining

Seeded samples were stained by immunofluorescence, after 3 h, 1 and 3 days. The samples were washed (PBS, twice), fixed (4% paraformaldehyde, 15 min), washed, permeabilized (0.1% Triton X, 10 min, at *RT*), then finally washed. A working solution was prepared, including fluorescent Phalloidin (red) (A12379, Invitrogen, USA), acting as an F-actin filament stain, and DAPI (4′, 6-diamidino-2-phenylindole) (blue), acting as a dsDNA stain. This working solution was added (40 min, shaking), then washed before the seeded samples were examined in a fluorescence microscope (Nikon Eclipse Ti, Tokyo, Japan).

#### Monitoring cell attachment and ECM deposition by SEM

At 3 and 14 days, seeded samples were prepared for SEM to observe cell attachment and ECM deposition, respectively. Samples were fixed in glutaraldehyde solution (2.5%, pH 7.2) for 30 min, then dehydrated through a graded series of ethanol solutions (70, 80, 95, and 100%) for 10 min/each. Dried samples were mounted on aluminum holders, sputter-coated with gold-platinum and examined by SEM using a voltage of 10 kV. The ECM contents were examined for the presence of Ca and P ions, identified by Energy Dispersive X-ray (EDX), at a working distance 5.5 mm.

#### Live/Dead staining assay

Seeded samples at 7 and 14 days were characterized for their cell viability, including intracellular esterase activity (green) and plasma membrane integrity using a LIVE/DEAD ® Viability/Cytotoxicity Kit for mammalian cells (Invitrogen). A stock solution of PBS containing Ethidium homodimer-1 (red, 2 µL mL^−1^) and Calcein AM (green, 1 µL mL^−1^) was prepared and vortexed. Seeded templates were washed (twice) by D-PBS (37 °C, 15 min) to remove remnant media and serum. The working solution (300 µL) was then added directly to cells (ensuring that all cells were covered with solution), before incubation (30 min, *RT*, shaking 100 RPM). The cells were then observed under fluorescence microscope at excitation/emission; Calcein AM = 494/517 nm, and Ethidium homodimer-1 = 528/617 nm. At least 10 Images were captured and stacked at 10 µm z-distance.

#### Lactate dehydrogenase (LDH) assay

LDH enzyme activity secreted in the culture medium was determined after 3, 7 and 21 days indicating the presence of apoptosis or toxicity of cells, thus evaluating indirectly the viability of the seeded cells. A calorimetric assay, LDH Assay Kit (ab102526, abcam), was used according to manufacturer’s protocol to measure the enzyme activity. To exclude the biological interference of FBS to the results, negative control samples (media including FBS, without cells) were set, and their absorbance optical density (OD) readings were subtracted from those of the test samples.

Only 10 µL from each sample (in duplicate, *n* = 4) was added to the reaction mix, and the output was measured immediately (within 5 min) at OD = 450 nm, on a multimode microplate reader (Varioskan™ LUX, VLBL00D0, Thermo fisher Scientific, Vantaa – Finland). LDH activity in the test samples was measured in a kinetic mode, every 3 min for a total of 30 min, protected from light. The results were calculated as *ΔA* = (*A*_*2*_*-A*_*1*_), where *A*_*1*_ is the OD at time 1 (*T*_*1*_ = 15 min) and *A*_*2*_ is the OD at time 2 (*T*_*2*_ = 21 min). The calculated *ΔA* was related to a standard curve to reveal the amount of reduced reagent (Nicotinamide adenine dinucleotide (NAD) to NADH), in nmol) generated by LDH during the reaction time (*ΔT*) (min). The total LDH activity of each sample was calculated as follows:

$$LDH\;activity=\frac{\mathrm{calculated}\;\mathrm{NADH}}{\mathrm\Delta\mathrm T\mathrm xV}(\mathrm{nmol}\;\min-1\mathrm{mL}-1)$$

where *V* is the original sample volume added to the reaction well (mL).

#### AlamarBlue assay

The metabolic activity of the cells was assessed by alamarBlue reagent (AlamarBlue HS, Invitrogen—Thermo Fisher Scientific, USA) (resazurin-based), that function as cell health indicator by using the reducing power of living cells to quantitatively measure viability. The reagent (30 µL) was added directly to cells in culture medium (300 µL) as directed by the manufacturer. The plates (*n* = 5) were incubated in a cell culture incubator (4 h, 37 °C) protected from direct light, and control (background) samples, containing culture media only, were used. From each well, 100 µL were aspirated (in duplicate) and added to 96 well plates to read immediate fluorescence (excitation at 560 nm, emission at 590 nm). The results were calculated by subtracting the background fluorescence from the fluorescence signal of the seeded templates.

#### Proliferation assay (DNA quantification)

DNA was quantified using a Quanti-iT PicoGreen® dsDNA assay kit (Invitrogen—Thermo Fisher Scientific, USA). At each timepoint, the seeded samples were stored in cell lysate solution (0.1% Triton X-100, 300 µL), frozen at -80 °C then thawed twice. Thawed samples (*n* = 5) were cut into pieces, put into 1.5 mL tubes (Eppendorf) together with the lysate solution, sonicated (10 min on ice), vortexed (1200 RPM, 10 s), then finally centrifuged for 1–2 min at 10,000 RPM. From the supernatant, 50 µL were aspirated and added to diluted Picogreen dye (in accordance with the manufacturer’s protocol). The intensity of fluorescence was measured at excitation/emission = 485/520 nm, and the cellular dsDNA content was calculated against a standard curve of a known concentration of DNA (µg mL^−1^), obtained by serial dilution.

#### Alkaline phosphatase (ALP) activity

The Alkaline phosphatase (ALP) activity was assessed as an indicator of osteogenic ECM secretion by the seeded cells. ALP was collected from cell lysate used in the DNA quantification assay (*n* = 5). *p*-Nitrophenyl phosphate (*p*NPP, Sigma) was added (1:1) to the thawed lysate solution to measure ALP expression. OD was measured at 405 nm at different time points (5, 10 and 15 min), and the results were normalized to cell number, determined by the proliferation assay.

#### Osteogenic gene expression analysis

The real-time quantitative polymerase chain reaction (RT-qPCR) technique was used to analyze the gene expression of seeded cells on different printed templates. RNA was extracted from samples at 7 and 21 days (*n* = 5) using a Maxwell® 16 LEV simplyRNA kit (Promega, Madison, WI, USA). The amount of RNA extracted was measured by spectrophotometry (Nanodrop ND-1000, Nanodrop Technologies, Wilmington, DE, USA). High-Capacity cDNA Reverse Transcription Kit (Applied Biosystems, Foster City, CA, USA), and SimpliAmp Thermal Cycler (Applied Biosystems) were used to synthesize cDNA. To detect the gene expression of the osteogenesis-related human genes, RT-qPCR was applied, using TaqMan Fast Universal PCR Master Mix (Applied Biosystems) and a StepOne™ RT-PCR System (Applied Biosystems). Each sample was assessed in duplicate, and the amplification efficiency of different genes (listed in Table S1) was determined relative to an endogenous control: glyceraldehyde-3-phosphate dehydrogenase (GAPDH) gene. The difference in threshold cycle value (*Ct*) was equal to *Ct* gene minus *Ct* GAPDH, while the mRNA in each sample was calculated using the comparative *Ct* (*Ct* gene—*Ct* control) value method. Data were analyzed by the 2^−Δ∆*CT*^ method and relative transcript levels of the PLATMC group were presented as fold change (in Log scale) relative to PCL.

#### Alizarin red staining

Assessment of osteogenic differentiation was based on ECM secretion and mineralization. The seeded samples were stained with Alizarin red (2% in dH_2_O at pH = 4.2) to measure calcium deposition on the printed templates. Samples (21 and 28 days) were fixed, washed, and kept drying. Enough stain was then added to cover each sample. The samples were then incubated (10 min), washed (dH_2_O, 5–6 times, overnight), followed by ethanol (70%) overnight, and then aspirated. The dried samples were examined by a stereo microscope (LEICA M205 C, Germany) with mounted microscope camera. The dye was extracted using cetylpyridinium chloride (100 mmol, 300 μL/sample, 4 h, *RT*) and quantified at OD = 544 nm using a microplate reader. After dye extraction, some samples were further monitored for any remaining attached mineralized matrix, by additional SEM qualitative analysis.

### In vivo characterization in rabbit model

The in vivo study comprised subcutaneous implantation and CBD models in New Zealand white (NZW) rabbits and was conducted at the Institute of Graduate Studies and Research (IGSR), Alexandria University, Egypt. The animal experiment protocol was reviewed and accepted by the institutional animal care and use committee (IACUC)—Alexandria University, approval no. AU14-191,013–2-5.

#### Subcutaneous implantation model surgery

Three adult male NZW rabbits (3–4 months old) were used in this study. 3D-printed PCL and PLATMC templates were implanted subcutaneously in the dorsal area in each rabbit (*n* = 3). The rabbits were anesthetized by Xylazine (10 mg kg^−1^, IM) and Ketamine (25 mg kg^−1^, IM). The dorsal area was widely shaved, to ensure a space of at least 5–6 cm between the samples. The area was then disinfected with povidone iodine. The incision lines were made on both sides of the dorsum, around 3 cm away from and parallel to the midline, followed by the subcutaneous dissection to form pouches to receive one of the pre-sterilized 3D-printed samples. The incision was then sutured and the position of each sample was also marked with cutaneous sutures. The samples were retrieved at 8 weeks post-implantation.

#### Calvarial defect model surgery

In total, eight skeletally-adult male NZW rabbits were used in this study. 3D-printed PCL and PLATMC templates were implanted in each defect (in random order). Using a trephine bur, two bone defects (Ø = 9 mm) were created bilaterally, on each rabbit calvarium, followed by the implantation of the prepared templates (2 mm thickness and 9 mm diameter). The surgical wound was closed in layers; the subcutaneous layer was closed with vicryl (3/0) resorbable sutures, while the skin layer was closed with silk (3/0) sutures. To prevent surgical site contamination, topical antibiotic (Gentamicin) was applied to cover the site.

Immediately after the surgery, a pain killer (diclofenac sodium, 5 mg kg^−1^, IM) was administrated daily (first 3 days after surgery). The silk sutures were removed after 1 week. The rabbits were euthanized after 4 and 8 weeks (*n* = 4 /time point/group). Collected bone samples were fixed, dehydrated, and processed for μCT and histology analysis.

#### Data collection and analysis

The μCT analysis was used to determine the amount of calcified bone formation within the implanted templates. This was followed by sectioning of samples and staining for histological examination and histomorphometric (quantitative) analysis. After histological examination, the samples were analyzed using NIS-Elements Software (Nikon, Japan).

For histomorphometric analysis, the total region of interest (ROI) was marked, from both edges of the template/defect, then the template area was calculated. The available defect area (ADA) was calculated as follows: ADA = Total ROI – template area. The sum of new bone area (NBA) within the defect was measured and total regenerated bone was calculated as NBA/ADA (%). The mean of the middle three sections in each sample was calculated, and the mean of each group (*n* = 4) was presented. For bone contact calculations, the entire length of new growing bone in direct contact with the template surface (bone contact length) was traced, while the total borders of new growing bone within the implanted templates (total bone boarders) were calculated. The values measured were expressed as a percentage of the bone contact length per total bone borders.

### Statistical methods and analysis

To carry out the statistical analysis, Prism software (GraphPad software, San Diego, CA, USA) was used and the results were expressed as group average ± standard deviations. For comparisons of mean values, t-test was applied. If the Levene’s test for variances was significant, the welch test assuming non-equal variances was applied. For the analysis over time, we applied multiple t-test with Holm-Šídák adjustment for multiple comparisons. The null hypothesis was rejected at p-value < 0.05.

## Results

### Comparison of printability and process parameters of PLATMC and PCL

Compared to PCL, the melting-extrusion of PLATMC was challenging and showed relatively uneven printing rates during processing through the extrusion-based printer head used with pneumatic pressure through a syringe. This required high pre-heating and relatively high printing temperatures: above 220 °C and around 195 °C, respectively (Table S2). However, both maintained reproducible structures closely related to their ideal design (Fig. 1a and b) and no intergroup differences were shown in the printability of PCL and PLATMC. On the other hand, there was no significant difference in printing-yield (gain) after the printing process (Fig. 1c), but the printed PLATMC revealed higher density than PCL (Fig. 1d).

### Physical advantages of PLATMC over PCL

**Fig. 2:**
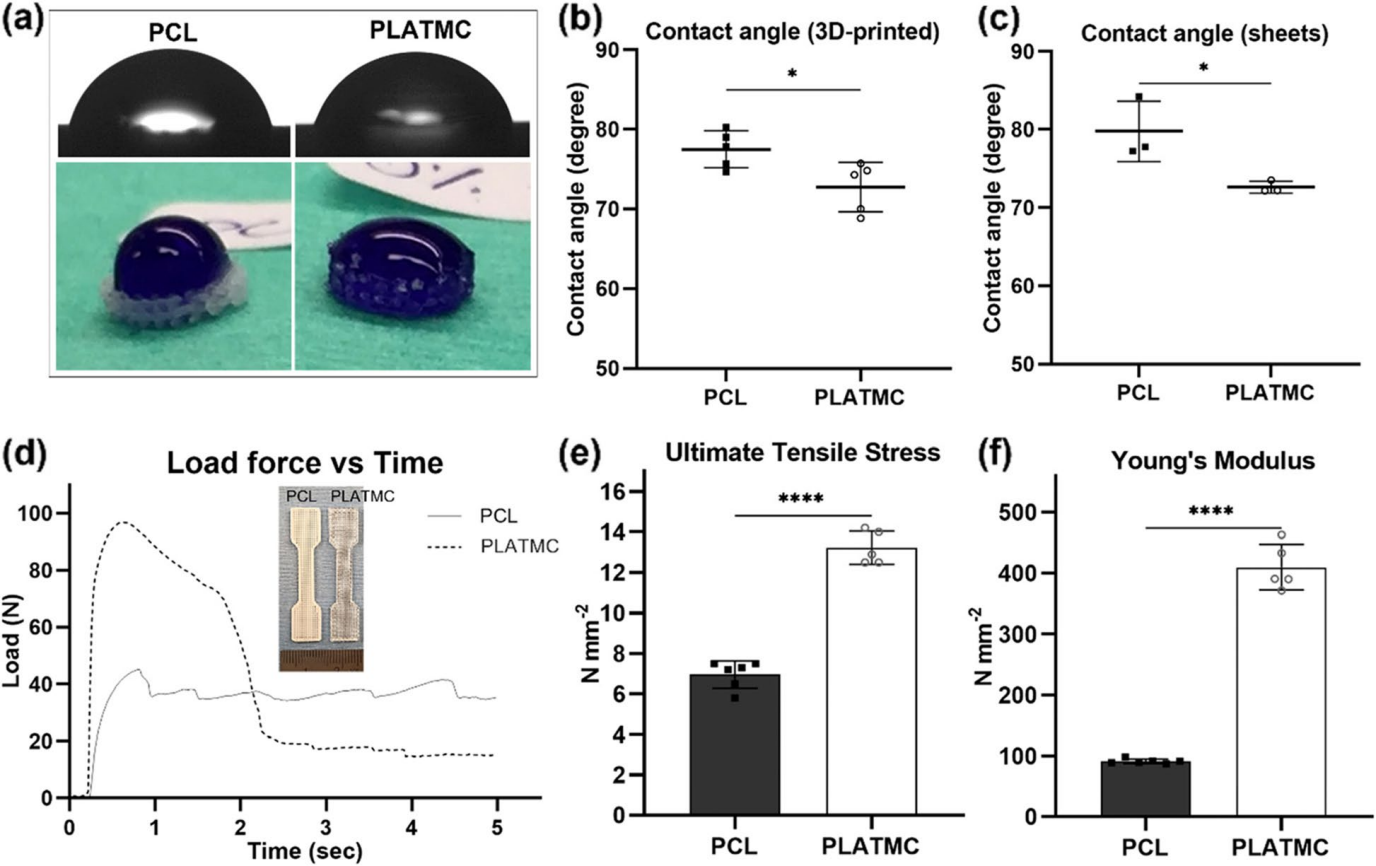
Physical characterization of the 3D-printed PCL and PLATMC, in terms of wettability and mechanical properties. **a** micrographs for contact angle measurement (top), and macroscopic images for the hydrophilic behavior using a drop of dye/water (bottom raw). **b** and **c** charts for the contact angle measurements of PLATMC versus PCL in 3D-printed (**b**) and casted sheet forms (**c**), respectively. **d** load force vs time curves, with inset photographs for the printed samples prepared according to ASTM-D638. **e** and **f** column charts of the mean ultimate tensile stress, and Young’s modulus, respectively. Note the significant higher wettability and tensile strength of PLATMC. * *p* > 0.0332, **** *p* < 0.0001

**Fig. 3:**
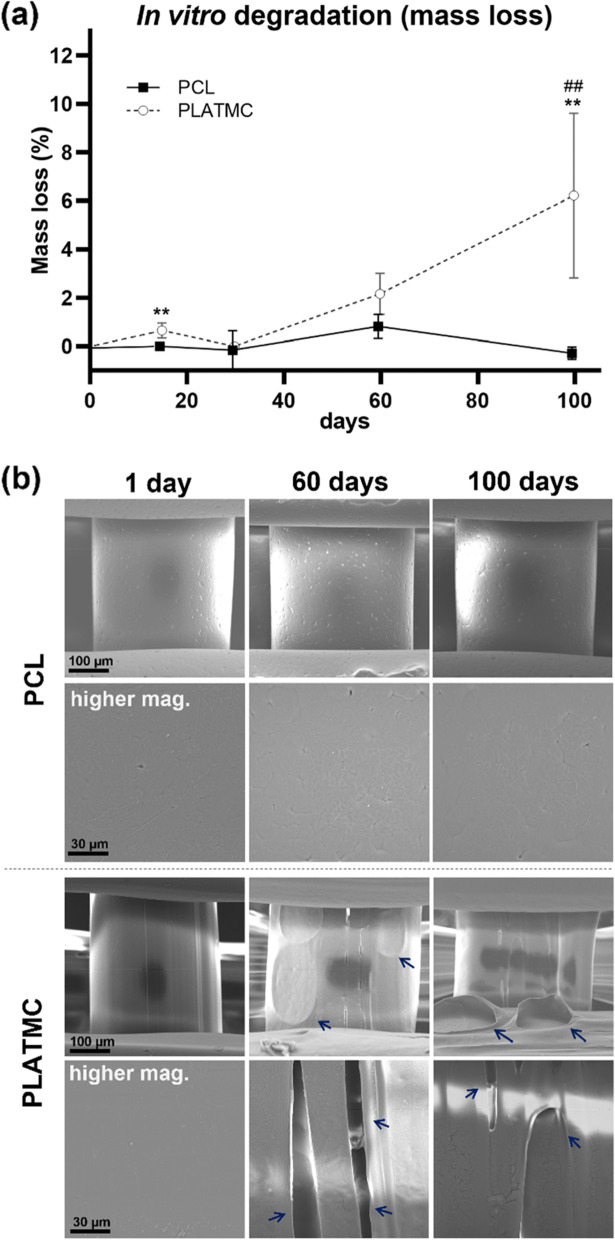
In vitro degradation of the 3D-printed PCL and PLATMC in PBS at 37 °C monitored up to 100 days. **a** line-graph for the mass loss quantification. Note the significant higher degradation rate of PLATMC compared to the undetectable degradation of PCL, while significance between each time point and the previous time point in the same group is marked with hash symbol (#), **p > 0.0021. **b** SEM micrographs for the printed templates at 1, 60 and 100 days, with signs of degradation marked with blue arrows

The wettability of PLATMC was significantly higher than PCL, with lower contact angles on the 3D-printed as well as the cast sheets (Fig. 2a - c). In addition, printed PLATMC revealed fourfold higher Young’s Modulus and twofold higher tensile strength than PCL (Fig. 2d - f). On the other hand, PLATMC showed slightly increasing degradation in vitro up to 60 days, with significant mass-loss (6.21% ± 3.39) recorded at 100 days (Fig. 3a) and showed obvious signs of degradation, including both bulk and surface erosion degradation (Fig. 3b). By comparison, PCL exhibited almost complete absence of degradation (0.28% ± 0.25).

### Osteoconduction in vitro and abundant ECM secretion on the PLATMC surface

**Fig. 4:**
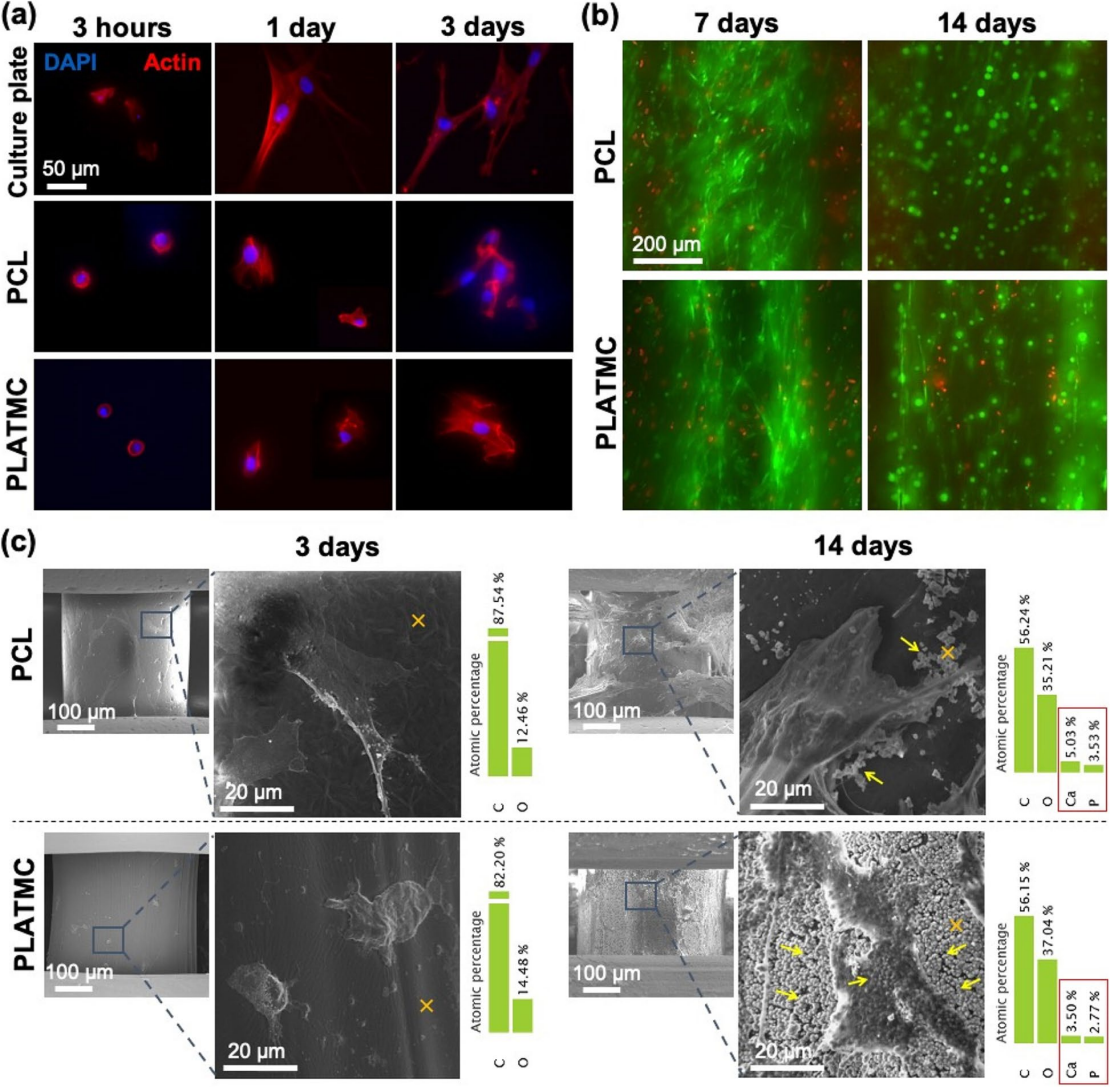
hBMSCs attachment, viability and ECM secretion on 3D-printed PCL and PLATMC: **a** microscopic images showing cytoskeleton immunofluorescence staining after 3 h, 1 day, and 3 days compared to culture plate surface (control); F-actin filaments stained by Phalloidin (red) and nuclei stained by DAPI (blue). **b** Live/dead stain for seeded cells after 7 and 14 days (z-stacked images). **c** SEM showing cell adhesion (3 days), and ECM deposition (14 days) and the corresponding EDX characterization to the substrate surface marked with (x). Note the abundant secretion of micron-sized globular accretions marked by YELLOW arrows on PLATMC compared to PCL (14 days), with their Ca and P contents characterized by EDX

No significant differences in initial seeding efficiency were noticed between PLATMC and PCL (Figure S1). Moreover, there were no observed differences in the early attachment of hBMSCs at 3 h and 1 day (Fig. 4a). However, at 3 days, the cells attached to PCL revealed higher proliferation and more spindle morphology, while stellate cellular morphology was observed on PLATMC, with noticeably enhanced F-actin polymerization, characterized by SEM and immunofluorescence, respectively (Fig. 4). However, live/dead stain disclosed no intergroup differences in cell viability up to 14 days (Fig. 4b).

The ECM secretion observed by SEM at 14 days on PLATMC was unique, with obvious abundant globular accretions of the cement line matrix, micron-size in diameter in the form of aggregated ECM vesicles (ECMVs), totally covering and adhering to the template surface (Fig. 4c). Whereas PCL groups showed inadequate ECM secretion, with considerably fewer numbers of rod-like shaped crystallites (2–4 µm in length). EDX characterization of the secreted ECM confirmed the presence of Ca and P ions in both groups, whereas the crystallites produced on PCL surfaces, revealed higher total atomic percentages of Ca and P than those presented within the globular accretions on PLATMC surfaces (Fig. 4c).

**Fig. 5:**
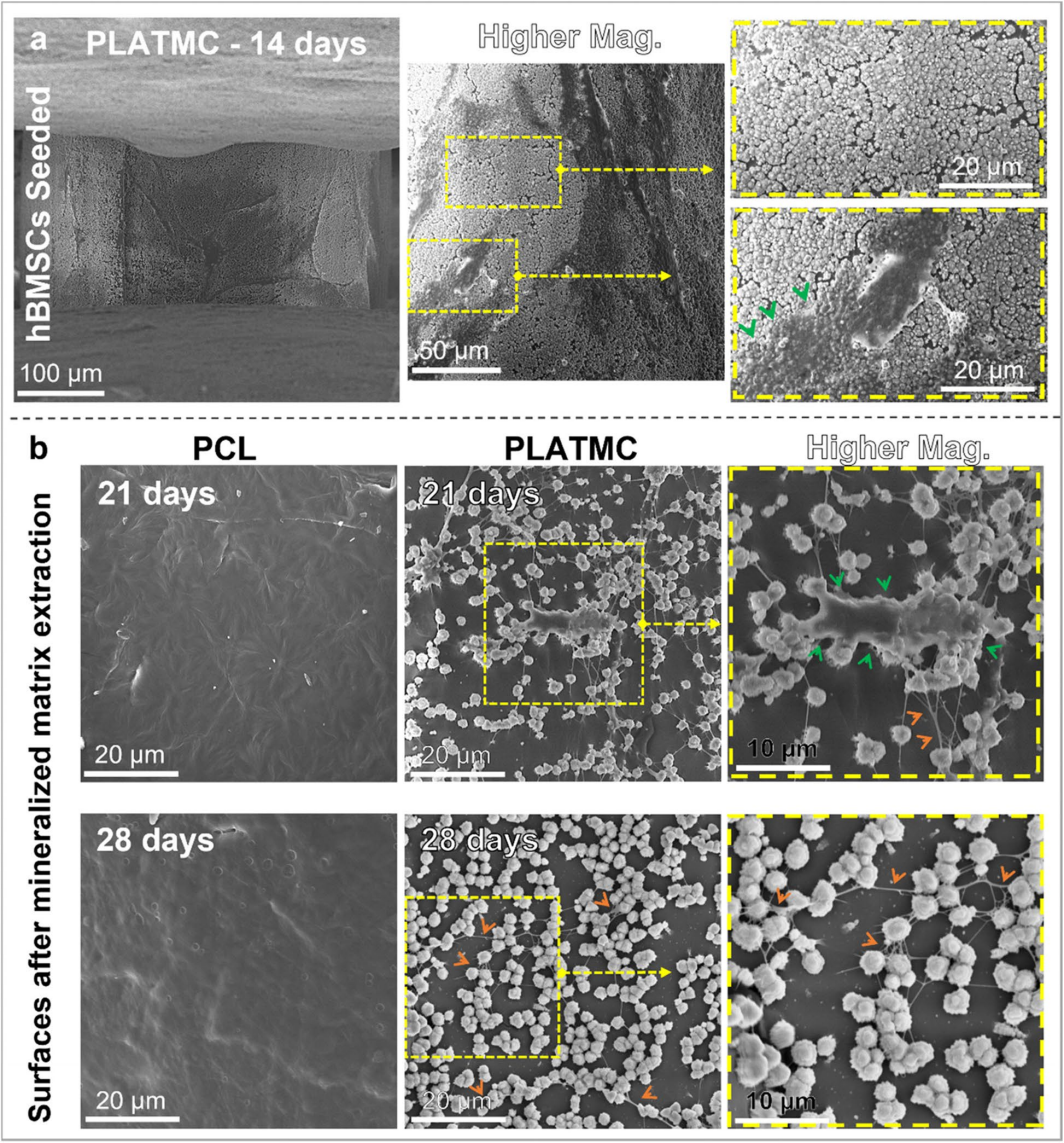
SEM micrographs analyzing the remarkable globular accretions of the cement line matrix, totally covering, and anchored to the surface of PLATMC templates. **a** General view of the globular layer secreted by seeded hBMSCs on the surface of 3D-printed PLATMC templates at 14 days. At higher magnifications, the surface is totally covered with globular (vesicular) layer in addition to layers of homogenous structural matrix on the top of the globular layer. **b** SEM micrographs of PCL and PLATMC samples, at 21 and 28 days after Alizarin red dye and mineralized ECM extraction, showing the persistent anchorage of globular accretions (1–2 µm diameter/each) to PLATMC surface, while no remaining matrix or cells were noticed on PCL. Not the cells/matrix anchored to the top of the globular accretions (Green arrowheads) and the connecting fibrillar collagen (ORANGE arrowheads)

The presented continuous layer of globular accretions of the cement line matrix covering PLATMC surface at 14 days was further characterized by SEM qualitative analysis, and spots of overlying cells and secreted structural matrix were shown on the top of the globular matrix layer (Fig. 5a). Furthermore, the samples characterized at 21 and 28 days after Alizarin red dye extraction (removal of mineralized matrix for quantification) revealed that globular accretions were totally adherent to PLATMC surfaces and were shown at the size of 1–2 µm in diameter/each. In addition, layers of remaining structural matrix were adherent on the top of the globular matrix (Fig. 5b). On the other hand, no remaining matrix or adherent globular accretions were found on PCL surface after dye extraction at 21 and 28 days (Fig. 5b).

**Fig. 6:**
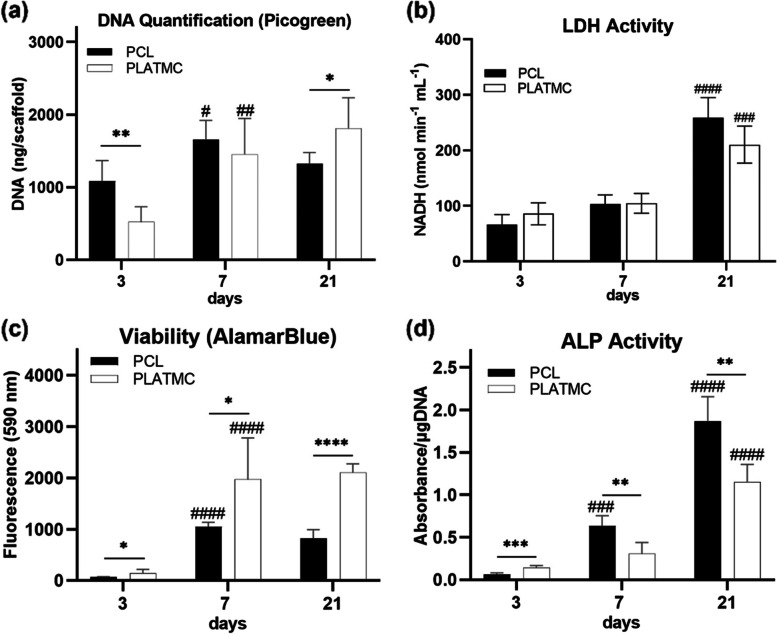
Quantitative analysis of cellular proliferation and activity of hBMSCs seeded on 3D-printed PCL and PLATMC at 3, 7 and 21 days, represented as column charts showing: **a** cell proliferation characterized by DNA quantification using Picogreen assay; **b** apoptotic tendency characterized by LDH activity assay; **c** cell metabolic activity characterized by alamarBlue assay; and **d** ALP activity. Note the higher proliferation rate and viability on PLATMC, while less ALP activity compared to PCL. Statistical significance between each time point and the previous time point in the same group is marked with hash symbol (#), while significance between the groups is marked with Asterisks (*) at *p* < 0.05; * *p* > 0.0332, ** *p* > 0.0021, *** *p* > 0.0002, **** *p* < 0.0001

The number of cells attached to the template surface detected through DNA quantification assay revealed earlier higher proliferation rate on PCL at 3 days. However, noticeable continuous proliferation was observed later only on PLATMC at 21 days (Fig. 6a). Meanwhile, the lactate dehydrogenase (LDH) activity assay revealed no intergroup differences in apoptotic tendency (Fig. 6b). On the other hand, the alamarBlue assay revealed significant metabolic activity of the seeded cells on PLATMC at all time points compared to PCL (Fig. 6c).

**Fig. 7:**
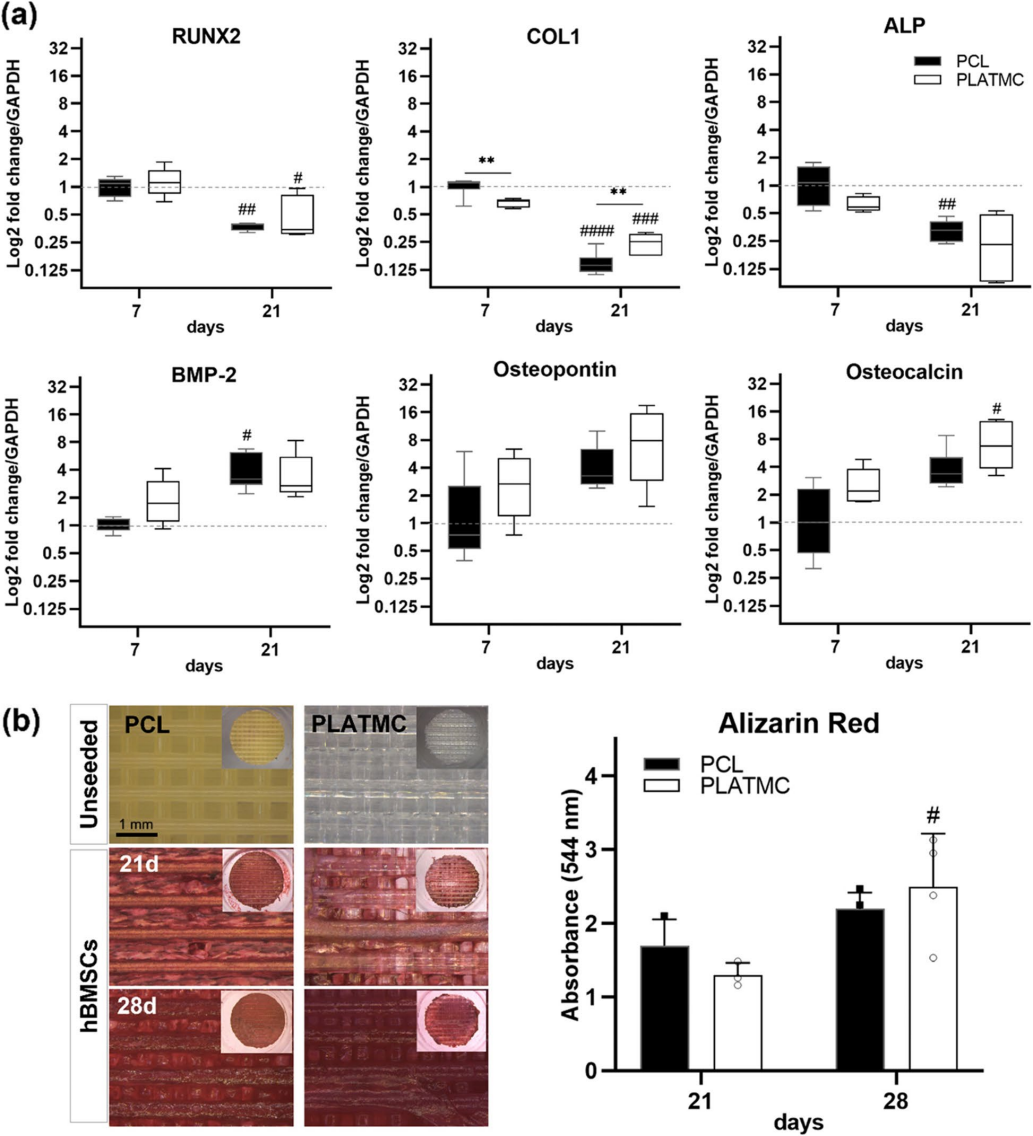
Osteogenic differentiation of hBMSCs seeded on 3D-printed PCL and PLATMC characterized by gene expression of osteogenic markers and Alizarin red staining. **a** box plots representing the gene expression of selected osteogenic markers at 7 and 21 days. **b** left-hand side shows micrographs of the mineralization stained by Alizarin red at 21 and 28 days, compared with unseeded templates (blank), while the inset pictures show the gross view. A column chart is plotted on the right-hand side representing the quantified optical density of the dissolved stain of each group subtracted from blanks (unseeded templates). Statistical significance between each time point and the previous time point in the same group is marked with hash symbol (#), while significance between the groups is marked with Asterisks (*) at *p* < 0.05; * *p* > 0.0332, ** *p* > 0.0021, *** *p* > 0.0002, **** *p* < 0.0001

PLATMC group underwent a significant increase in ALP activity as early as 3 days compared to PCL. However, it was of interest to note that PCL exhibited significant boost in ALP activity at 7 and 21 days (Fig. 6d). This was also apparent at the gene level, where PCL group at 7 days revealed higher ALP expression together with statistically significant enhanced collagen type I (COL1) expression (Fig. 7a). Instead, the other osteogenic markers were normally expressed by both groups; early markers (RUNX2 and BMP-2) at 7 days and late markers (Osteopontin and Osteocalcin) at 21 days (Fig. 7a). In addition, Alizarin red staining at 21 days showed equivalent mineralization in both groups, while significant active mineralization continued only in PLATMC at 28 days (Fig. 7b).

### PLATMC promotes new bone formation in vivo through contact osteogenesis

**Fig. 8:**
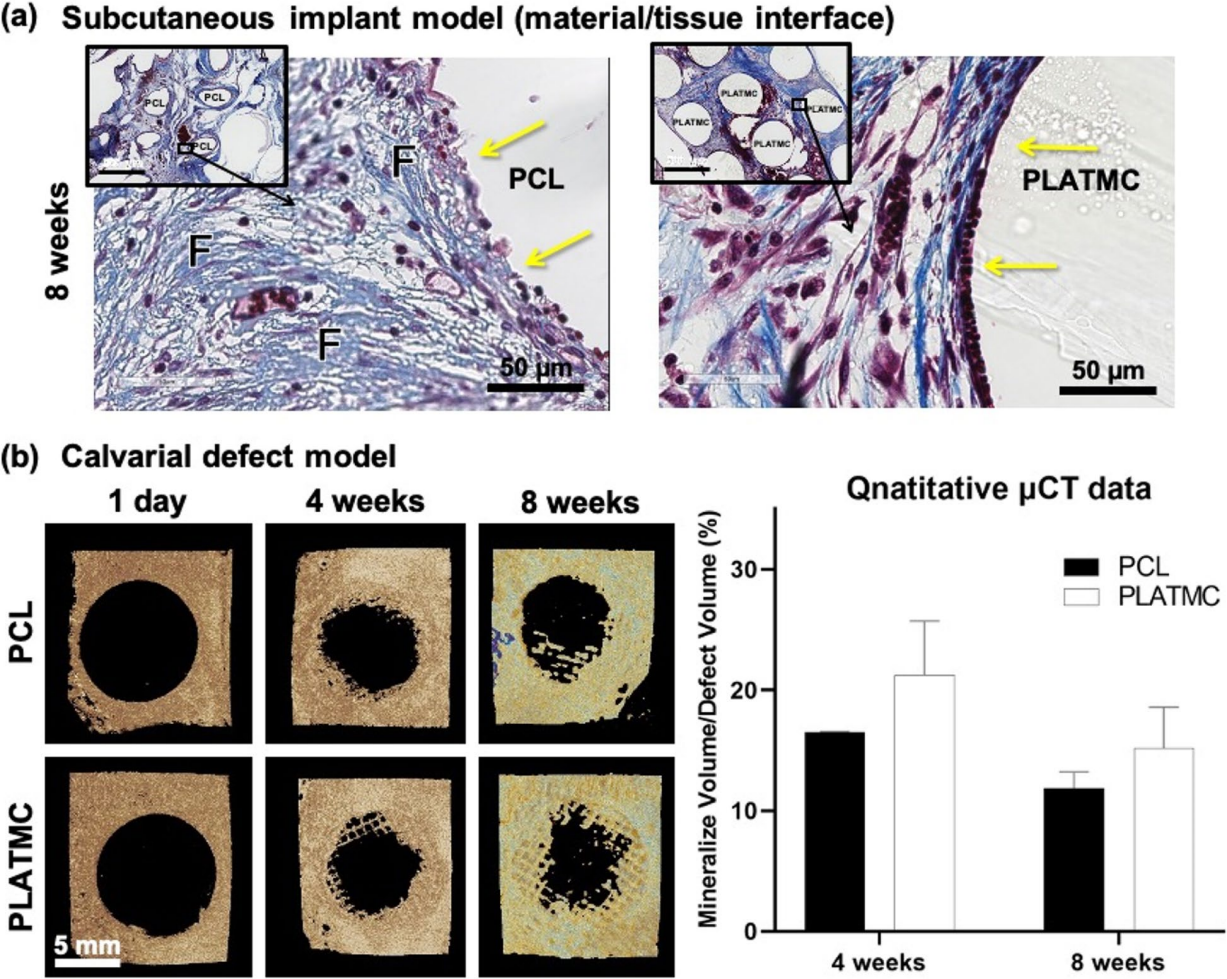
Summary of the outcomes from in vivo implantation of 3D-printed PCL and PLATMC templates (in rabbits); in subcutaneous model and in calvarial defect model. **a** representative histological micrographs of the subcutaneously implanted templates focusing on the material/tissue interface at 8 weeks as indicated by YELLOW arrows (scale bar = 50 µm), stained with Massons’ trichrome, while the inset figures represent the overall view at lower magnification (scale bar = 500 µm); (F) represents fibrous connective tissues. **b** µCT reconstructed pictures of the calvarial defect model at 4 and 8 weeks, while a bar chart is plotted on the right-hand side representing their quantified mineralized volume/total defect volume (*n* = 4 /group/timepoint)

Within the implanted 3D-printed templates in the subcutaneous model (8 weeks), there were no signs of ectopic bone formation or mineralization in either group. The observed biomaterial/tissue interface at PLATMC indicated a highly cellular loose connective tissue interface, with few mononuclear inflammatory cells, and fewer macrophages (Fig. 8a). On the other hand, PCL exhibited a much denser connective tissue interface, more abundant macrophages and thin-walled vascular invasion with large areas of bleeding, despite considerable variation from one area to another.

In the CBD, it was observed that the bone growth towards the defect center was clearly following the scaffold strands from around the defect margins. As seen in the µCT results (Fig. 8b), the best rate of mineralized bone ingrowth occurred on PLATMC templates as early as 4 weeks (21.2% ± 4.5), but less observed mineralized bone ingrowth at 8 weeks (15.2% ± 3.3). Nevertheless, within the defect area at PCL templates, smaller amount of mineralized bone was quantified that revealed (16.4% ± 0.8) and (11.9% ± 1.3) at 4 and 8 weeks, respectively.

**Fig. 9:**
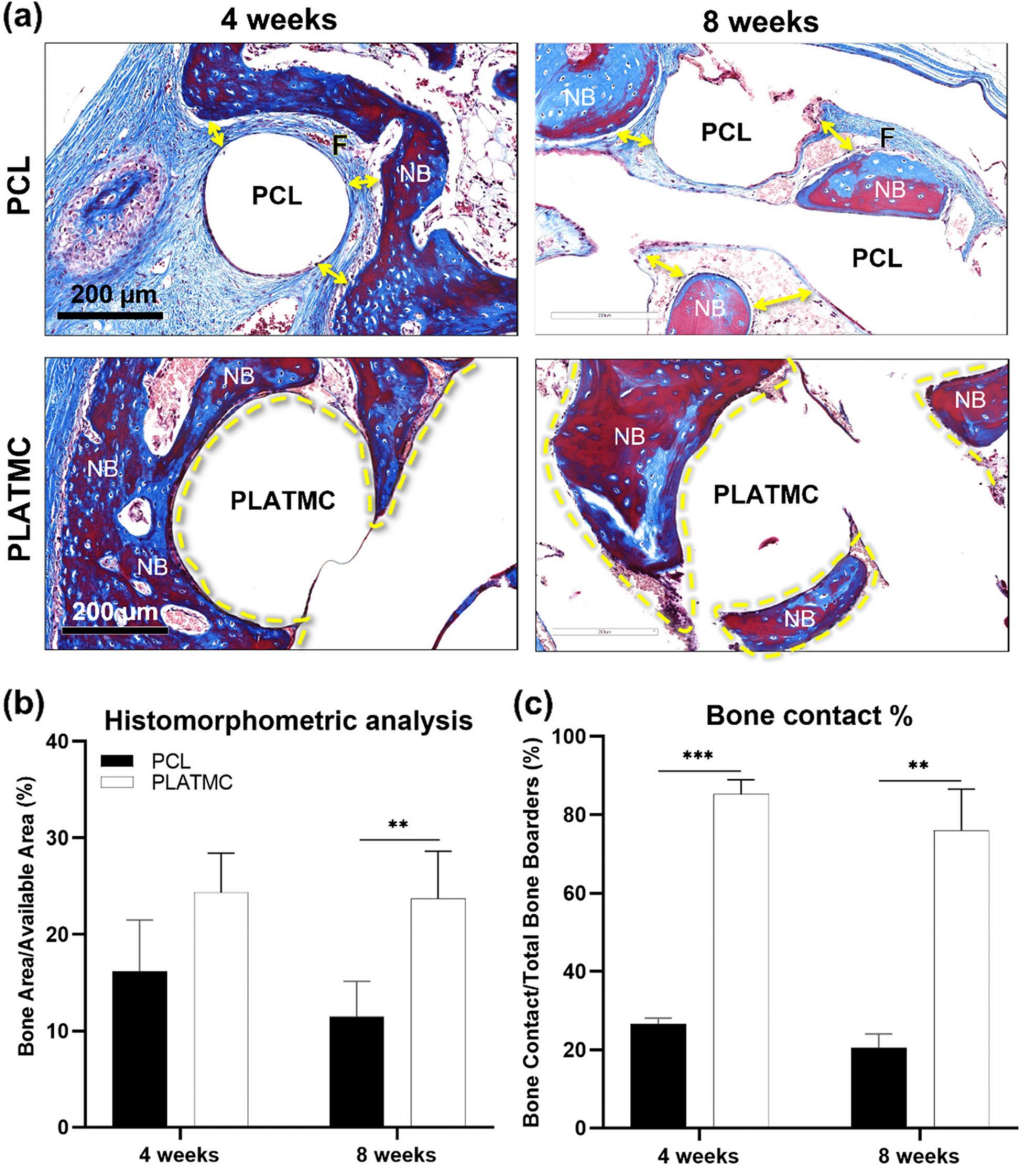
Summary of the histological outcomes of 3D-printed PCL and PLATMC templates implanted in the calvarial defect model (in rabbits). **a** histological micrographs (stained with Masson’s trichrome) at 4 and 8 weeks (scale bar = 200 µm) showing the interface of new bone with template strands. Note the direct contact (contact osteogenesis) of the new formed bone on PLATMC. **b** and **c** represents the quantitative histomorphometric analysis and bone contact (%) calculation, respectively. (F) represents fibrous connective tissues; (YELLOW dashed Line) represents areas of contact osteogenesis (present only at PLATMC); (NB) represents areas of new bone; (YELLOW double arrow) represents the characterized gap (fibrous connective tissue) at material/tissue interface (present only at PCL)

On the other hand, histological examination (Fig. 9a) disclosed characteristic contact osteogenesis of de novo bone on PLATMC strands, at both 4 and 8 weeks, whereas on PCL strands a fibrous connective tissue interface was usually seen separating the growing new bone from PCL surface. Quantitative histomorphometric analysis of histological sections disclosed greater new bone area at PLATMC with (24.3% ± 4.1) and (23.7% ± 4.9), at 4 and 8 weeks, respectively, compared to PCL templates (16.1% ± 5.2) and (11.4% ± 3.6). A statistical intergroup significance was disclosed at 8 weeks (*p* = 0.0299) (Fig. 9b). in addition, calculations of the bone contact (%) showed significance on PLATMC (85.3% ± 3.6) and (75.9% ± 10.6) which was 2.5 to threefold higher than PCL (26.6% ± 1.4) and (20.6% ± 3.5) at 4 and 8 weeks, respectively (Fig. 9c). Thus, PLATMC exhibited noticeable contact osteogenesis while PCL revealed apparent distance osteogenesis, with minimum new bone contact.

## Discussion

Particularly for polymer-based templates used for BTE, 3D-printing is a promising alternative to the methods previously used to fabricate 3D porous templates, while improving the mechanical resistance of the structure. For BTE, 3D-printing has showed satisfactory outcomes [26]. It can reproducibly create customized templates with specific or complex anatomic shapes, with highly porous structure and superior interconnectivity [30]. In the present study, PLATMC was selected for investigation because of recent reports of its favorable physical and biological properties in soft tissue applications [31, 32]. The study comprised extensive characterization, to test the osteoconductivity of PLATMC for potential BTE applications. Direct comparison was made with FDA approved, 3D-printed medical grade PCL [14, 33].

Printability is defined as the capability of polymer to form and maintain reproducible 3D-templates using a defined printing technique. This affects the structure of the printed templates, in relation to their ideal design, and consequently affects their mechanical and biological properties [34, 35]. In the present study, the printability of both PCL and PLATMC was very close to their ideal design. However, PLATMC required little real-time adjustments in the printing temperature and speed while printing. This variation in parameters could be related to the recently reported significant loss of molecular weight of PLATMC during printing [31]. The calculated tensile mechanical performance of PLATMC was close to the previously reported ranges [36] and markedly better than the tensile properties of PCL. The same applies to the reported bulk degradation of PLATMC, attributed to leaching out of water-soluble oligomers and low molecular-weight polymers [36].

The seeded cells on PCL showed earlier proliferation by fluorescence microscopic images and DNA quantification at 3 days. However, with regard to in vitro osteogenic differentiation, the seeded hBMSCs on PLATMC showed stellate-like morphology, with enhanced F-actin polymerization (3 days), and were normally differentiated and committed to the osteogenic lineage, as evidenced by ALP activity at 3 days and by the expression of RUNX2 and BMP-2 at 7 days [37, 38]. This was in addition to the steady proliferation rate, as shown by DNA quantification at 21 days on PLATMC, and noticeable higher metabolic activity revealed by alamarBlue assays at all time points.

The abundant globular matrix layer observed on PLATMC at 14, 21 and 28 days covering and adherent on its surface, was found to be a remarkable distinction from PCL. This justified the subsequent in vitro active mineralization, and in vivo contact osteogenesis seen with PLATMC. This was on agreement to the previously described studies that pointed to ECMVs and globular accretions as the key structure deposited by osteoblasts as a cement line matrix, interdigitating with osteoconductive implanted/substrate materials, above which the mineralizing collagen matrix can be seen [6, 39].

In the reviewed literature, secreted ECMVs, usually about 200 nm in diameter, were defined as membrane-invested globular structures which concentrate calcium (Ca) and Phosphate (P) ions, released by budding from the surface of active osteoblasts [40]. Moreover, ECMVs usually aggregate, with noncollagenous proteins including osteopontin, and increase in size, creating larger mineralized globular accretions, around 1 µm in diameter [41]. In consequence, mature osteoblasts should lay down COL1 (known as the structural matrix), together with ALP secretion, to initiate mineralization in alkaline environment [40, 42].

Globular accretions were considered the dominant feature of the mineralizing nodules, before the deposition of bone-like matrix in osteoblast cultures [41], adipocyte-derived differentiated osteoblasts [43], and on other BTE substrates [44]. This was also explored in the current study, after Alizarin red dye and matrix extraction from seeded PLATMC templates at 21 and 28 days, that disclosed how the globular cement line matrix was quite persistent and firmly anchored to PLATMC surface.

In contrast, the higher ALP activity at 7 and 21 days in addition to the higher expression of ALP and COL1 at 7 days led to the observation of mineralized crystallites on the surface of PCL as early as 14 days. The mineralized crystallites appeared as rod-like shaped structures, bigger than the globular accretions observed on PLATMC, and with higher Ca and P contents, indicating existing mineralization, i.e. CaP crystallization. However, these crystallites were scarce and accompanied by significantly limited cellular metabolic activity, as evidenced by alamarBlue assays at 7 and 21 days. This in turn revealed a reasonable amount of mineralization, detected by Alizarin red staining at 21 and 28 days.

Meanwhile, as expected, mineralization as high as seen on PCL was observed on PLATMC at 21 days, due to the earlier noticed reduction in ALP activity and COL1 expression on PLATMC at 7 days, compared to PCL. Nevertheless, unlike PCL later at 28 days, PLATMC group exhibited significantly continued active mineralization which led to boosted mineralization, detected by Alizarin red staining. This could be due to the markedly higher secretion of ALP and expression of osteopontin and osteocalcin at 21 days than that at 7 days.

In literature, PCL is reported to act through a Smad-dependent BMP pathway [45], which enhances cell differentiation and ALP activity, but usually downregulates self-renewal of the preosteoblast as the differentiation potential increases [46]. It could be assumed from the data currently shown, that PLATMC induces a different pathway, the TGF-β signaling pathway, to promote the early osteoblastic lineage commitment of hBMSCs, through selective MAPKs and Smad2/3 pathways [47]. TGF- β signaling was found to inhibit ALP activity and osteoblast mineralization to promote proliferation through a MAP3K-dependent pathway [48]. In addition, when templates were coated with natural-derived ECM [49], or osteogenic growth peptide [50] a MAPK/ERK signaling pathway was reported to stimulate much higher osteogenic differentiation and activation of hBMSCs. However, this needs further investigation and confirmation for PLATMC.

Because of the absence of osteogenic cues required for osteogenic lineage differentiation, the subcutaneous implantation of 3D-printed templates of PCL and PLATMC did not result in ectopic bone formation. Instead, a dense fibrous connective tissue interface was typically seen with PCL, corresponding to the foreign body reaction to implanted PCL reported in previous studies [51]. In contrast, much less fibrous-related foreign body reaction was observed in the host response to PLATMC, but rather a loose connective tissue interface with high cellular infiltration was shown. On the other hand, a recent study by our group reported ectopic mineralization on cell-free constructs of 3D-printed PLATMC and human platelet lysate hydrogels (HPLG), when implanted subcutaneously in nude mice after 4 and 8 weeks [52]. Although HPLG has some advantages, no organized bone-like tissue or entrapped cells were observed.

In the CBD model, where the environment is rich in osteogenic signals, a potent osteoconduction and greater amount of new bone ingrowth were observed on PLATMC. The quantified new bone detected by µCT showed advantage for PLATMC compared to PCL, with no statistically significant intergroup differences. However, on histological examination, marked amount of new bone ingrowth was observed on PLATMC at 8 weeks and definite contact osteogenesis of the new formed bone to PLATMC surface was observed at both 4 and 8 weeks.

In the current study, the active mineralized matrix production and contact osteogenesis on PLATMC surface were presented only in vitro and in the calvarial defect model, where osteogenic supplements and signals are presented. Hence, this is typically presented by osteoconductive surfaces but no osteoinductive properties were shown, as demonstrated by the subcutaneous implantation model. It should be noted that the contact osteogenesis observed on 3D-printed PLATMC has not been reported previously for any synthetic polymer used for BTR, or even for blended polymers with osteoconductive bioceramics [53, 54]. These interesting findings could be related to the observed in vitro results, including stimulation of surrounding cells to attach, proliferate and secrete globular cement line matrix directly onto the PLATMC surface, only in osteogenic supplement medium. Such defined physical and biological findings favor the application of PLATMC as a BTE template which combines both biodegradation and osteoconductivity.

## Conclusion

Compared to PCL, PLATMC templates exhibited markedly superior wettability, mechanical and degradation properties. The study disclosed biological advantages favoring the application of 3D-printed PLATMC templates for bone tissue engineering.

The seeded cells exhibited initial faster proliferation as early as 3 days on PCL, while on PLATMC they exhibited earlier osteogenic differentiation and higher metabolic activity. Abundant secretion of globular accretions of the cement line matrix was shown totally covering the PLATMC surface as early as 14 days and disclosed as active mineralization process in vitro up to 28 days of culture. This was also reflected in vivo as early as 4 weeks, when new bone ingrowth was observed with evident contact osteogenesis. As a synthetic co-polymer, PLATMC was unique in its ability to activate osteoconduction and contact osteogenesis on its surface.

## Supplementary Information

**Additional file 1.**

## Data Availability

Not applicable.

## Abbreviations

ALP: Alkaline phosphatase
BTE: Bone tissue engineering
CBD: Calvarial bone defect
COL1: Collagen type I
ECM: Extracellular matrix
ECMVs: Extracellular matrix vesicles
EDX: Energy Dispersive X-ray
hBMSCs: Human bone marrow-derived mesenchymal stem cells
LDH: Lactate dehydrogenase
OD: Optical density
PCL: Polycaprolactone
PLATMC: Poly(lactide-co-trimethylene carbonate)
PTMC: Poly(trimethylene carbonate)
ROI: Region of interest
SEM: Scanning electron microscopy
