# Supplementary material for: Contact osteogenesis by biodegradable 3D-printed poly(lactide-co-trimethylene carbonate)
Source: Biomater Res. 2022 Oct 10;26:55. doi: 10.1186/s40824-022-00299-x (PMC9552430; doi:10.1186/s40824-022-00299-x)

**Supporting Information**

**Contact Osteogenesis by Biodegradable 3D-printed Poly(lactide-co-trimethylene carbonate)**

Mohamad Nageeb Hassan^*^, Mohammed Ahmed Yassin, Ahmed Maher Eltawila, Ahmed Emad Aladawi, Samih Mohamed-Ahmed, Salwa Suliman, Sherif Kandil, and Kamal Mustafa^*^

Table S1: List of genes assessed in the current study.

| Gene and code |  | Name |  | Role |
| --- | --- | --- | --- | --- |
| GAPDH Hs02758991_g1 |  | Glyceraldehyde-3-phosphate dehydrogenase |  | House-keeping gene |
| Runx-2 Hs01047973_m1 |  | Runt-related transcription factor 2 |  | Early osteogenic marker  (for osteoblast differentiation) |
| ALPL  Hs01029144_m1 |  | ALP; Alkaline phosphatase, liver/bone/kidney |  | Early to intermediate osteogenic marker |
| COL1A2 Hs00164099_m1 |  | COL1; Collagen, type I, alpha 2 |  | Early to intermediate osteogenic marker |
| BMP-2 Hs00154192_m1 |  | Bone morphogenetic protein-2 |  | Early to intermediate osteogenic marker |
| SPP1 Hs00959010_m1 |  | Osteopontin |  | Late osteogenic marker |
| BGLAP Hs01587814_g1 |  | Osteocalcin; Bone gamma carboxyglutamate protein |  | Late osteogenic marker |

Table S2: Printing parameters of PCL and PLATMC

| Group | Pressure | Temperature ^a)^ | Printing speed | Printing Time | Feed |
| --- | --- | --- | --- | --- | --- |
|  | [bar] | [^o^C] | [mm sec^-1^] | [min] | [g] |
| PCL | 8.4 | 110 | 1.6 | 360 | 3.5 |
| PLATMC | 8.0 | 195 | 2.0 - 5.0 | 85 | 3.0 |

^a)^ All polymers were pre-heated for 15 min before printing at 15-25 ^o^C beyond the actual recorded printing temperature.

Figure S1: (a) morphology of the seeded hBMSCs at passage 4 (just before seeding) and (b) the quantification of seeding efficiency on PCL and PLATMC (b).


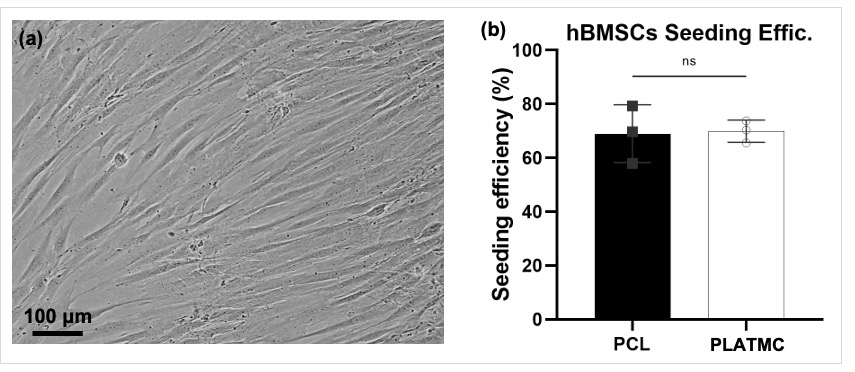

Supplement: Supplementary file 1 — Additional file 1. [file 40824_2022_299_MOESM1_ESM.docx]
